# Association between prognostic nutritional index and prognosis of patients receiving coronary artery bypass grafting surgery: a systematic review and meta-analysis

**DOI:** 10.3389/fcvm.2026.1673038

**Published:** 2026-02-19

**Authors:** Yashan Lei, Wei Wang, Changjun Hua, Ya Chen

**Affiliations:** Department of Rehabilitation Medicine, The First Affiliated Hospital of Chongqing Medical and Pharmaceutical College, Chongqing, China

**Keywords:** coronary artery bypass grafting, meta-analysis, mortality, prognostic nutritional index, systematic review

## Abstract

**Objectives:**

To conduct the first systematic review and meta-analysis to assess the association between prognostic nutritional index (PNI) and prognosis of patients receiving coronary artery bypass grafting surgery (CABG).

**Methods:**

We conducted a systematic literature search via PubMed, Embase, Web of Science, and Cochrane until March 2025, for studies that evaluated the association between PNI and prognosis of patients receiving CABG. All-cause mortality and acute kidney injury (AKI) were the primary outcomes. Odds ratios (OR) and 95% confidence intervals (CI) were used for data pooling. In addition, sensitivity analysis and subgroup analysis were performed to evaluate the stability of the results and potential sources of heterogeneity. All data analyses were conducted using Review Manager 5.4 and STATA 15.1 software.

**Results:**

A total of 11 studies including 11,444 patients were included in the meta-analysis. The results showed that, compared with the low PNI group, the high PNI group had a significantly lower all-cause mortality rate (OR: 0.81; 95% CI: 0.72, 0.90) and a significantly lower risk of acute kidney injury (OR: 0.82; 95% CI: 0.77, 0.86). Sensitivity analyses confirmed that the associations between PNI and all-cause mortality and AKI were stable.

**Conclusions:**

PNI can effectively predict postoperative all-cause mortality and AKI in patients undergoing CABG. Considering the inevitable heterogeneity and potential publication bias in this article, more large-scale, multicenter, prospective cohort studies are needed in the future to assess the predictive value of PNI for prognosis after CABG and to identify its influencing factors.

**Systematic Review Registration:**

https://www.crd.york.ac.uk/PROSPERO/view/CRD420251077959, PROSPERO CRD420251077959.

## 1 Introduction

Coronary atherosclerotic heart disease (CAD) refers to a condition in which the coronary artery lumen becomes narrowed or occluded due to atherosclerosis. In recent years, due to changes in lifestyle, its incidence has increased among younger populations, and the number of deaths caused by it has ranked first worldwide. In 2020, the Institute for Health Metrics and Evaluation at the University of Washington (1) conducted a study on a total of 369 diseases across 204 countries or regions. The study concluded that ischemic heart disease, particularly CAD, is now the primary cause of mortality and disability worldwide. It found that from 1990 to 2019, the number of patients with CAD increased year by year, ranking first (1). Coronary artery bypass grafting (CABG) is a crucial treatment for severe CAD, particularly in cases of left main or three-vessel disease (2). The evaluation of risk related to CABG requires a multifactorial approach, including cardiac surgical risk factors (such as age, body mass index, medical history, and cardiac function) and CABG-specific factors (such as the location and extent of coronary artery disease, graft selection, and surgical technique) (3, 4). Given the inherent complexity of this procedure, it is essential to conduct a thorough evaluation to ensure the best possible outcome.

The prognostic nutritional index (PNI) was introduced by Japanese researchers in 1984 to evaluate the preoperative nutritional condition, surgical risk, and potential postoperative complications in surgical patients (5). The index was refined based on the research conducted by the University of Pennsylvania Hospital in the United States (6). It is calculated using albumin and lymphocyte count, and its calculation formula is serum albumin (g/L) + 5× peripheral blood lymphocyte count (×10^9^/L). This formula makes PNI simple to obtain and calculate in clinical practice. Previous studieshave shown that adequate nutritional support can significantly reduce the occurrence of postoperative complications and mortality, indicating that individuals with higher PNI values are less likely to experience long-term adverse outcomes following surgery.

In recent years, with the continuous exploration of PNI, more evidence have emerged supporting its broad clinical application. Beyond its role in cancer, PNI has also demonstrated predictive value in a variety of other diseases, particularly in assessing the prognosis and severity of cardiovascular and cerebrovascular conditions, which is receiving special attention (7). A meta-analysis by Zhang et al. (8), which comprised studies published before 2022, found that malnutrition assessed by PNI can serve as an independent predictor of mortality and major adverse cardiac events (MACE) in CAD patients. However, the study by Zhang et al. did not focus on the prognostic value of PNI for CAD patients undergoing CABG (8). Since its publication, several large-scale clinical studies have investigated the association between PNI and outcomes in CABG patients, but their conclusions have been inconsistent (9–11).

Therefore, this aim of this study is to evaluate the prognostic value of PNI in patients undergoing CABG through systematic literature retrieval and meta-analysis, and provide the latest evidence-based outcome for the development of a prognostic model based on PNI for post-CABG patients.

## 2 Methods

### 2.1 Literature search

In accordance with the PRISMA 2020 (Preferred Reporting Items for Systematic Reviews and Meta-Analyses) guidelines, this meta-analysis was prospectively registered in the PROSPERO database (CRD420251077959). To identify relevant studies examining the link between the PNI and clinical outcomes in patients undergoing CABG, we systematically searched PubMed, Embase, Web of Science, and the Cochrane Library up to March 2025. The literature search was based on the use of specific keywords, including: “Prognostic nutritional index”, “PNI”, and “Coronary Artery Bypass”, etc. The specific search terms and strategy applied in PubMed are outlined below: [“Coronary Artery Bypass”(Mesh) OR “Coronary Artery Bypass” OR “Coronary Artery Bypass Grafting” OR “CABG” OR “Aortocoronary Bypass”] AND (“prognostic nutritional index” OR “PNI”). In addition to the database search, the reference lists of all included studies were manually examined to find any additional relevant publications. Two reviewers conducted the article selection and eligibility assessment separately. Any differences identified during the screening were addressed through mutual discussion. A detailed overview of the search approach is provided in Supplementary Table S1.

### 2.2 Inclusion and exclusion criteria

Studies were considered eligible if they met the following criteria: (1) the study design was a cohort study, randomized controlled trial, or case-control study; (2) the population included patients undergoing CABG; (3) the prognostic value of the PNI was assessed in relation to CABG outcomes; (4) at least one clinical outcome—such as all-cause mortality or AKI—was reported; and (5) adequate data were available to calculate odds ratios (ORs).

Studies were excluded if they were protocols, unpublished manuscripts, or non-original works (such as letters, abstracts, comments, corrections, or replies). Reviews and studies lacking adequate data for analysis were also excluded.

### 2.3 Data extraction

Two reviewers independently performed data extraction, and in case of discrepancies, a third author was consulted to resolve the issue. The following data were collected from each eligible study: name of the first author, year of publication, country in which the study was conducted, study design, patient population, sample size, age, gender, PNI cutoff value, and adjusted OR from multivariate analysis. When essential data were missing or unclear, the corresponding authors were contacted to obtain the full dataset, if available.

### 2.4 Quality evaluation

The methodological quality of the included cohort studies was evaluated using the Newcastle-Ottawa Scale (NOS) (12), with scores ranging from 7 to 9 indicating high-quality studies (13). Two reviewers independently performed the quality assessment, and any discrepancies were resolved through discussion.

### 2.5 Statistical analysis

The meta-analysis was performed using Review Manager software (version 5.4.1). Adjusted ORs and their 95% CIs were calculated to synthesize the data. To assess heterogeneity across studies, the chi-squared test (Cochran's Q) and the I^2^ statistic were employed (14). A *p*-value from the *χ*^2^ test below 0.1 or an *I*^2^ value exceeding 50% was interpreted as indicating substantial heterogeneity. A random-effects model (DerSimonian and Laird methods) was applied to calculate the pooled adjusted ORs for all outcomes. In addition to retaining the DL method for estimating *τ*^2^, the Hartung–Knapp–Sidik–Jonkman (HKSJ) method is used to further correct the confidence interval of the pooled effect size. Subgroup analyses were conducted where sufficient data were available to investigate possible confounding factors. Additionally, sensitivity analyses were carried out to determine the impact of each individual study on the overall OR for each outcome. To evaluate potential publication bias, funnel plots were generated using Review Manager 5.4.1, and Egger's regression test (15) was performed in Stata 15.1 (Stata Corp, College Station, Texas, USA). A *p*-value of less than 0.05 was considered indicative of significant publication bias.

## 3 Results

### 3.1 Literature retrieval, study characteristics, and baseline

**Figure 1:**
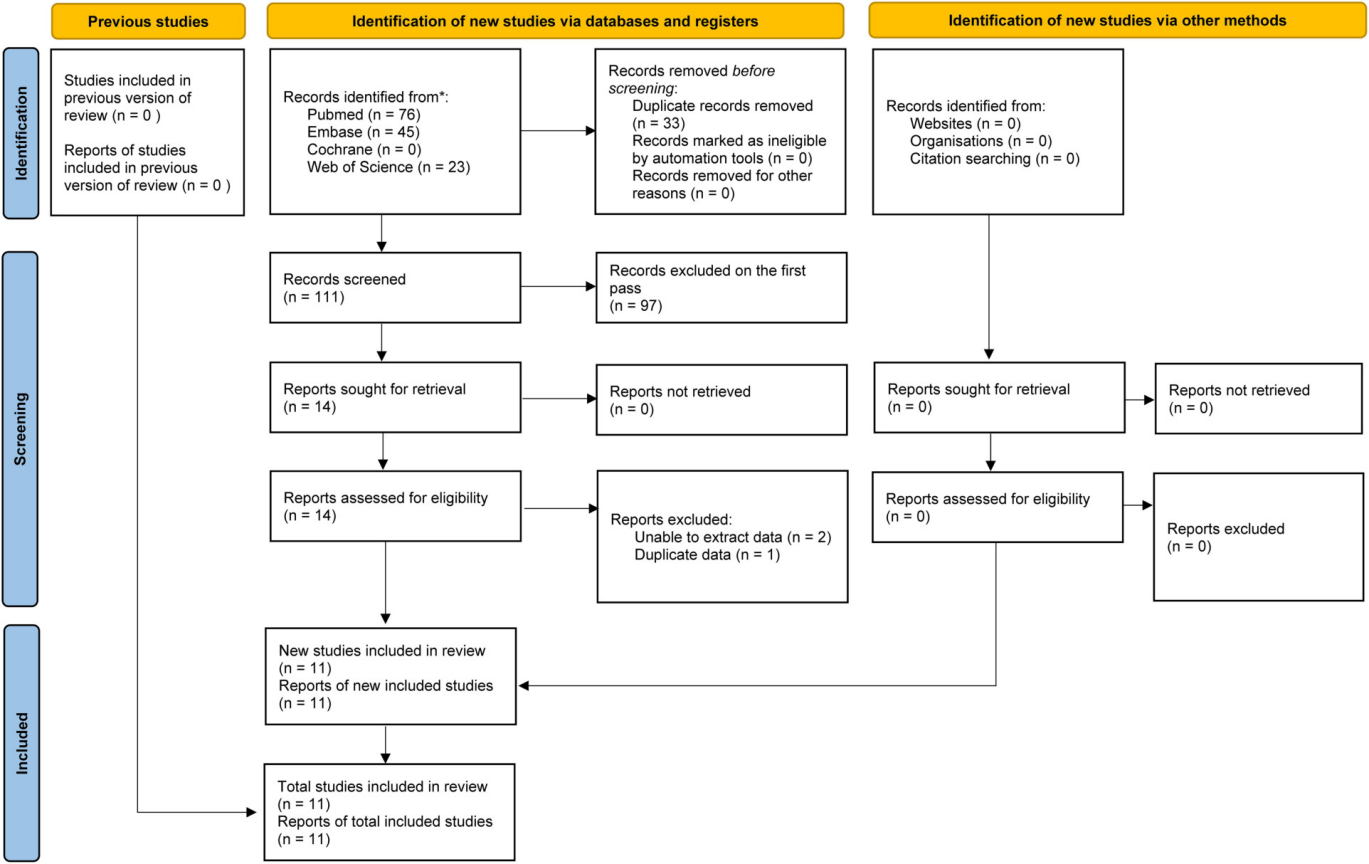
Flowchart of the systematic search and selection process.

Figure 1 illustrates the flowchart detailing the study selection process. A total of 144 relevant records were identified through a systematic search of PubMed (*n* = 76), Embase (*n* = 45), Web of Science (*n* = 23), and Cochrane Library (*n* = 0). After removing duplicates, 111 unique records were screened for by title and abstract. Ultimately, 11 cohort studies encompassing 13 comparative groups. The meta-analysis included 11,444 individuals (11, 16–23). Table 1 provides a summary of the characteristics and quality evaluations of these studies.

**Table 1:** Characteristics and quality assessment of included studies.

Author	Region	Study design	Population	No. of patients	Gender		Mean/median Age	PNI cut-off	NOS score	Adjusted factors
					Male	Female				
Aykut 2022	Turkey	Retrospective	Patients who underwent on-pump coronary artery bypass grafting	455	374	81	62	48	9	Intraoperative dobutamine use, Preoperative sCr, Preoperative hemoglobin
Bao 2024	China	Retrospective	Older patients underwent CABG	1,007	736	271	73.46	48	7	Gender, congestive heart failure, chronic pulmonary disease, SOFA score, MBP, preoperative hematocrit, BUN, postoperative creatinine
Cui 2023	China	Retrospective	Patients undergoing coronary artery bypass grafting surgery	879	690	189	65	48.1	7	Age, Sex, Weight Height, CHD, Ccr, LVEF, Valvular disease Obesity, Preoperative AF
Demirci 2024	Turkey	Retrospective	Patients with ST elevation myocardial infarction required emergent CABG	131	111	20	58	44.9	7	Age, LVEF, Glucose, Albumin
Dolapoglu 2019	Turkey	Retrospective	Patients with normal serum creatinine levels undergoing CABG	336	252	84	63.3	46.5	7	CRP, Positive inotropic usage, Diabetes Mellitus
Gucu 2021	Turkey	Retrospective	Patients with insulindependent diabetes who underwent on-pump CABG	254	151	103	62.7	42.9	8	Hypertension, Total perfusion time, Inotropic support Blood product use, Pre-creatinin, HbA1c
Keskin 2018a	Turkey	Retrospective	Patients with CAD undergoing CABG	644	538	106	63	NA	7	Age, sex, and BMI
Keskin 2018b	Turkey	Retrospective	Patients with CAD undergoing CABG	644	538	106	63	NA	7	Age, sex, and BMI
Keskin 2018c	Turkey	Retrospective	Patients with CAD undergoing CABG	644	538	106	63	NA	7	Age, sex, and BMI
Koyuncu 2024	Turkey	Retrospective	Patients who underwent CABG	239	171	68	69.5	39.1	7	Age, Diabetes Mellitus, Hemoglobin, Albumin, Lymphocyte, Platelet Index
Kwon 2022	Korea	Retrospective	Patients who underwent off-pump coronary artery bypass grafting	2,149	1,672	477	64	NA	7	Age, History of stroke, Infection, Aspirin
Liu 2025	China	Retrospective	Older adults (70 to 90 years) who underwent initial CABGonly surgery	1,173	848	325	73.32	44.425	7	Age, gender
Sun 2025	China	Retrospective	Patients who underwent isolated CABG	2,889	2,208	681	64.67	44.025	7	Age, gender, Body mass index, Smoking, Diabetes.

### 3.2 All-cause mortality

**Figure 2:**
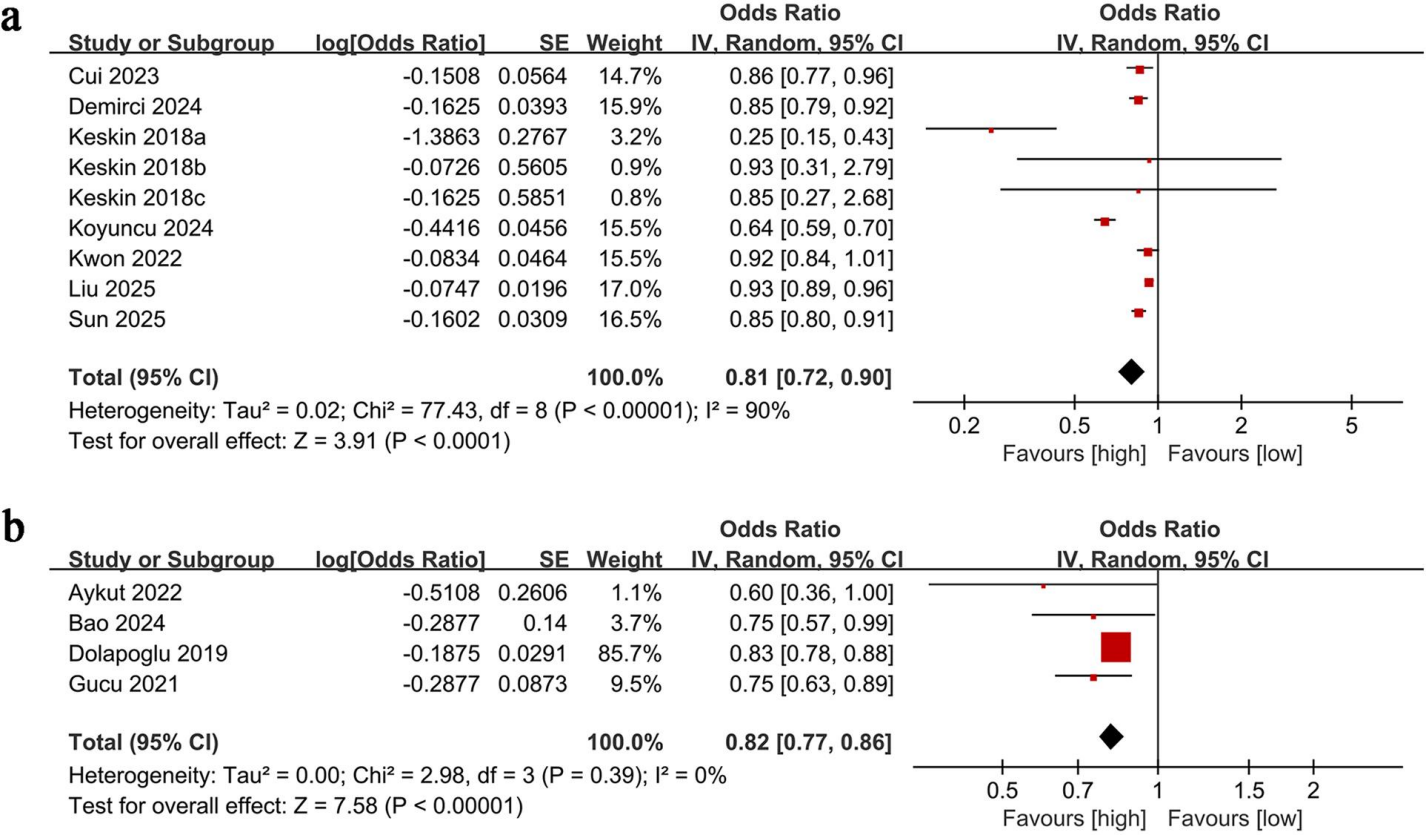
Forest plots of **(a)** all-cause mortality and **(a)** AKI.

All-cause mortality data were extracted from nine studies. Our analysis showed a significantly lower all-cause mortality rate in the high PNI group compared to the low PNI group (OR: 0.81; 95% CI: 0.72, 0.90; *P* <0.0001). However, significant heterogeneity was observed across the studies (*I*^2^ = 90%, *P* <0.00001) (Figure 2a). The statistical difference remained significant even after correction using the HKSJ method (Adjusted OR: 0.81; 95% CI: 0.68, 0.97).

**Figure 3:**
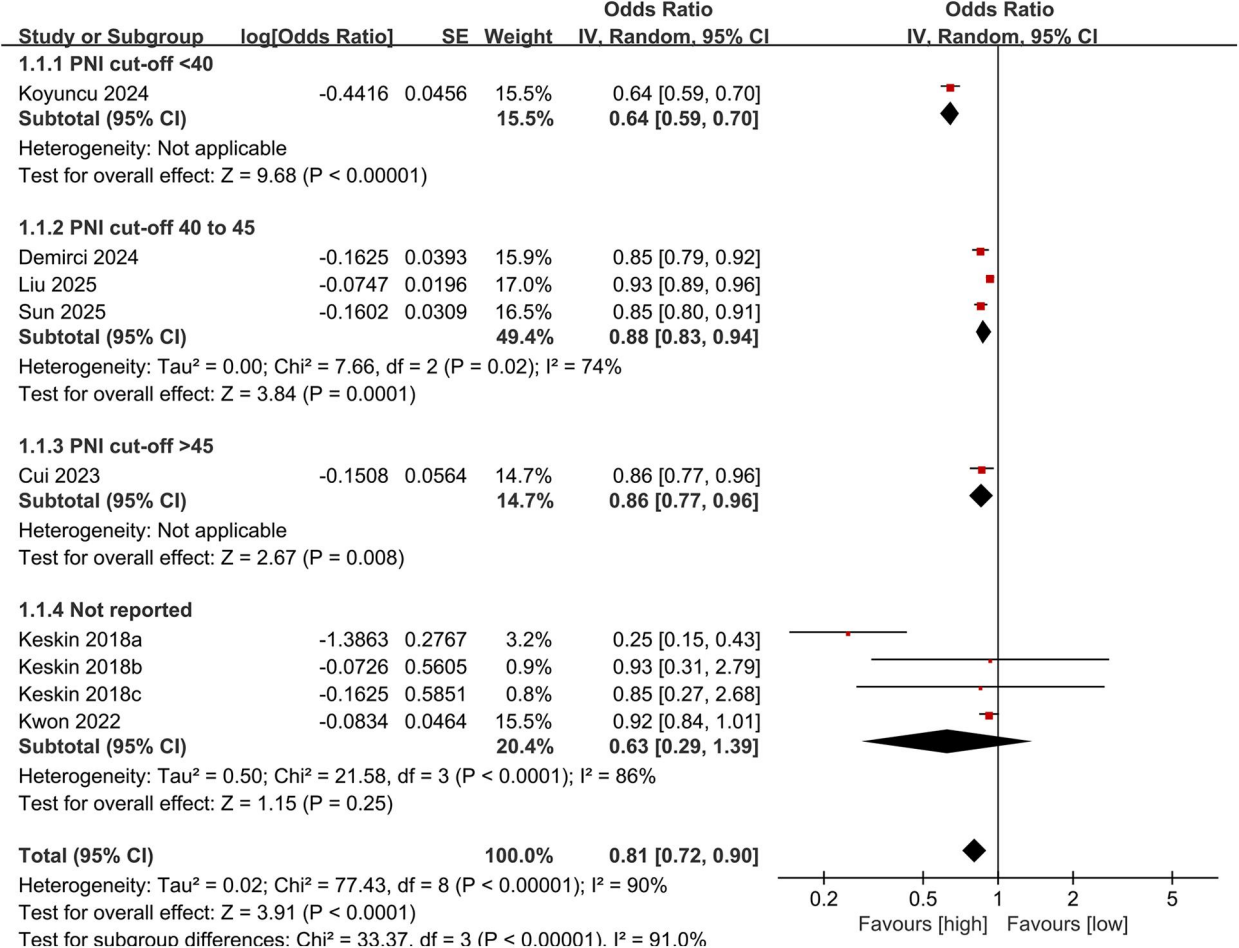
Subgroup analysis of all-cause mortality based on PNI cut-off.

A subgroup analysis of all-cause mortality based on PNI cut-off values was also performed. The results showed that the link between PNI and lower all-cause mortality persisted significant in all defined subgroups with cut-off: <40 (OR: 0.64; 95% CI: 0.59, 0.70), 40–45 (OR: 0.88; 95% CI: 0.83, 0.94), and >45 (OR: 0.86; 95% CI: 0.77, 0.96). However, in studies that did not clearly report the cut-off, no significant association was observed (OR: 0.63; 95% CI: 0.29, 1.39) (Figure 3). Besides, heterogeneity of all-cause mortality was significantly reduced in the subgroup with a cut-off of 40–45.

### 3.3 AKI

AKI outcomes were analyzed based on data from four studies. The meta-analysis showed a significantly lower risk of AKI in the high PNI group compared to the low PNI group (OR: 0.82; 95% CI: 0.77, 0.86; *P* <0.00001). No significant heterogeneity was observed (*I*^2^ = 0%, *P* = 0.39) (Figure 2b). The statistical difference remained significant even after correction using the HKSJ method (Adjusted OR: 0.81; 95% CI: 0.76, 0.88).

### 3.4 Publication bias and sensitivity analysis

**Figure 4:**
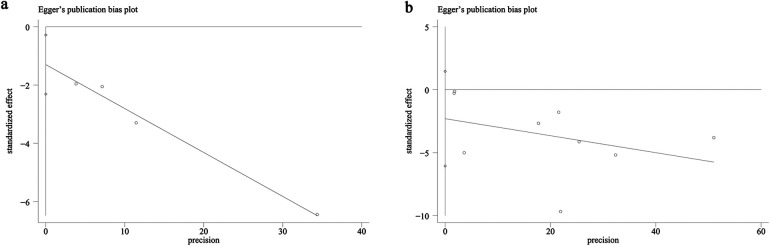
Egger's test plots of **(a)** AKI and **(b)** all-cause mortality.

**Figure 5:**
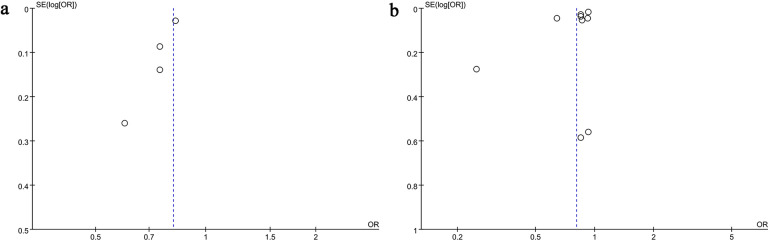
Funnel plots of **(a)** AKI and **(b)** all-cause mortality.

**Figure 6:**
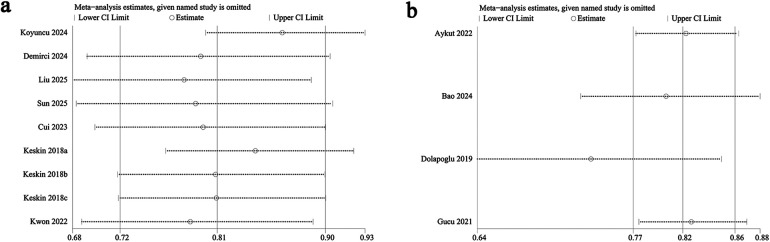
Sensitivity analysis of **(a)** all-cause mortality and **(b)** AKI.

The potential for publication bias in both all-cause mortality and AKI outcomes was assessed through the use of funnel plots and Egger's regression analysis. Egger's test (*P* = 0.031, Figure 4a) and funnel plots (Figure 5a) detected a significant publication bias for AKI. However, neither the Egger's test (*P* = 0.190, Figure 4b) nor the funnel plot (Figure 5b) detected publication bias for all-cause mortality. Additionally, a sensitivity analysis was conducted for both all-cause mortality and AKI outcomes to evaluate the influence of each individual study on the overall OR. By sequentially excluding each included study, we found that the overall ORs remained stable for both all-cause mortality (Figure 6a) and AKI (Figure 6b), indicating the results were not unduly influenced by any single study.

## 4 Discussion

This study found that PNI was significantly linked to postoperative all-cause mortality and the risk of AKI in individuals undergoing CABG. Higher PNI levels were linked to lower risks of both all-cause mortality and AKI. Sensitivity analyses confirmed the stability and reliability of these findings, indicating that the results were not substantially influenced by any single study. However, a notable publication bias was observed for the AKI outcome, which could influence the robustness of the findings to some extent. We speculate that regional selection bias may be one of the main causes of publication bias, because the literature included in this study are all studies in Asia, mainly in China and Turkey, which also needs to be further resolved through international multicenter studies. In addition, significant heterogeneity is also an issue that cannot be ignored in this study. Based on this, we performed subgroup analysis according to the PNI cut-off value. The results indicate that the significant heterogeneity of all-cause mortality may be partly attributed to the PNI cut-off. Future studies involving multiple PNI cut-off gradients are expected to solve this problem.

A previous meta-analysis by Zhang et al. (8) similarly concluded that malnutrition, as assessed by PNI, could functions as an independent predictor of mortality and MACE in individuals with CAD. However, since only one study in our meta-analysis reported the association between PNI and MACE, data pooling was not feasible. Notably, Zhang et al. did not focus on the treatment of CAD patients, the predictive value of PNI for postoperative AKI in CAD patients was not reported (8), which is also one of the advantages of this study.

Although this meta-analysis preliminarily explored the association between different PNI cutoff values and prognosis through subgroup analysis and found a consistent protective trend across subgroups, the heterogeneity of PNI cutoff values ​​in current studies (ranging from 39.1 to 48.1) remains a significant source of outcome heterogeneity. Optimizing the PNI cutoff value is crucial for its widespread clinical application. Future research should utilize statistical methods such as receiver operating characteristic (ROC) curve analysis and the maximum Yangon index to determine the optimal cutoff value for specific CABG populations (e.g., different ages, comorbidities, or surgical procedures) in large, multicenter cohorts, and establish a dynamic threshold system to accommodate individualized risk assessment. Furthermore, the setting of clinical thresholds needs to balance sensitivity and specificity. For example, using PNI < 40 as a high-risk cutoff may be suitable for identifying groups with a significantly increased risk of death, while PNI 40–45 can serve as a moderate-risk warning, thus providing a clear and operational stratification basis for preoperative nutritional intervention and postoperative management.

The reason why PNI is significantly related to all-cause mortality and AKI after CABG surgery may be, to some extent, because the nutritional status of patients is actually closely related to their own immune response. When the body has an immune response, a large amount of proinflammatory mediators and cytokines will be produced (24). The inflammatory response caused by this induces protein breakdown, increases the resting metabolic rate, and increases the body's demand for protein, resulting in poor nutritional status of patients. These conditions put critically ill patients at high risk of malnutrition, and the complications that follow are significantly increased. Inflammation is a key factor in the development of CAD (25). In recent years, with the continuous deepening of research on coronary atherosclerosis, inflammatory response (26) has become another new theory of its pathological mechanism. This theory believes that inflammatory response can affect the progression of coronary atherosclerosis in three aspects: promoting the progression of stable atherosclerosis, causing plaque instability and thus triggering acute cardiovascular events, and damaging the myocardium and participating in ventricular remodeling after cardiovascular events. The study of Alparslan Kurtul et al. directly revealed that PNI can reflect the long-term immune response and nutritional status of patients with myocardial infarction (27).

In addition, serum albumin and lymphocytes, two key components of PNI, are closely related to inflammatory responses. Serum albumin not only has the physiological function of maintaining plasma osmotic pressure and capillary permeability (28), but is also a ligand for many endogenous and exogenous compounds. Related studies have shown that serum albumin is a negative acute phase reactant (29). After serum albumin synthesis decreases, the acute phase inflammatory response begins rapidly (30). When serum albumin is at physiological concentrations, it can selectively inhibit the activation of vascular cell adhesion factor and intercellular adhesion factor in human endothelial cell adhesion molecules induced by tumor necrosis factor, thereby exerting an anti-inflammatory effect (31). When serum albumin levels decrease, proinflammatory cytokines are activated, further promoting the development, formation and rupture of coronary artery atherosclerotic lesions (32).

On the other hand, lymphocytes are the smallest white blood cells, mainly produced by lymphoid organs, and are the main players in the body's immune function (33). More and more studies (34) have shown that a decrease in peripheral blood lymphocyte counts is closely linked to the development of premature coronary artery disease. This may be because the process of lymphocyte redistribution from peripheral blood to lymphoid tissues (35) will induce compensatory proliferation of antigen-experienced T cells, thereby increasing plaque load and causing acute plaque rupture. At the same time, a study by Lana Fani et al. (36) showed that lymphocytes mobilize anti-inflammatory cells such as regulatory T cells to the body's atherosclerotic sites in specific immunity, secrete transforming growth factor-β and interleukin-10 to inhibit inflammation and stabilize plaques, and provide a protective response to cardiovascular and cerebrovascular disease lesions. Therefore, sufficient evidence shows that PNI can predict adverse outcomes after CABG surgery in CAD patients by displaying inflammatory response pathways.

While this meta-analysis confirms the prognostic value of PNI in CABG patients, it is important to note that the included studies exhibit potential differences in surgical techniques (e.g., off-pump vs. cardiopulmonary bypass CABG) and regional surgical practices. Off-pump CABG may indirectly influence the association between PNI and postoperative complications (e.g., AKI) by reducing cardiopulmonary bypass-related systemic inflammatory responses and hemodilution effects. Furthermore, differences in patient baseline characteristics, perioperative nutritional support strategies, and postoperative rehabilitation protocols across different regions (e.g., Asia vs. Europe and the Americas) may introduce clinical heterogeneity, partially explaining the high heterogeneity observed in this study. Future research requires more rigorously designed prospective studies to conduct in-depth analyses of specific surgical technique subgroups or different geographical populations to clarify the precise prognostic utility of PNI in these specific contexts.

Certain limitations associated with our analysis must be acknowledged. First, owing to the nature of clinical research, some of the analyzed studies are retrospective. It is widely recognized that possible confounding factors and risk of bias are the biggest disadvantages of retrospective studies. Furthermore, the included studies are predominantly from Asia, specifically China and Turkey, with limited data from Europe and the Americas. As a result, the extent to which these results can be applied to populations in other regions remains unclear. Besides, although this study preliminarily explored heterogeneity through subgroup and sensitivity analyses, a significant methodological limitation was the failure to perform meta-regression analysis to systematically quantify the impact of potential effect-modifying factors (such as mean patient age, severity of underlying disease, or surgical procedure) on the observed pooled outcomes. Due to the relatively limited number of included studies and inconsistencies in reporting key covariates, the statistical power required for reliable meta-regression analysis was insufficient, limiting our ability to delve deeper into the precise sources of high heterogeneity. Therefore, this study primarily provides general evidence of the association between PNI and prognosis, but struggles to precisely elucidate the patterns of variation in this association across different clinical subgroups. Lastly, the results of this study are subject to unavoidable heterogeneity and selection bias, which have not been fully explained. Therefore, caution is advised when interpreting the predictive value of PNI for the risk of postoperative all-cause mortality and AKI in CABG patients. Despite the limitations discussed above, no previous meta-analysis has examined the prognostic significance of PNI following CABG. The results obtained from this analysis underscore the significance of PNI levels in the clinical management of CAD patients. They also highlight the potential for developing a more valuable prognostic model based on inflammatory indicators like PNI, aimed at improving the long-term prognosis and quality of life for CAD patients following CABG.

## 5 Clinical implementation strategies

This meta-analysis confirms that PNI is an effective predictor of prognosis in CABG patients, but its clinical translation requires a systematic implementation strategy. First, as a cost-effective indicator based on routine blood tests, PNI can be integrated into routine preoperative assessment procedures to quickly identify high-risk patients, thereby enabling early nutritional intervention and personalized perioperative management. Standardized operating procedures must be clearly defined during implementation: including uniform blood collection time, standardized testing methods, and the establishment of institution-specific PNI cutoff reference ranges. Second, clinical pathways should incorporate multidisciplinary collaboration: the nutrition department can develop intensive nutritional support programs for patients with low PNI; the cardiac surgery team can adjust surgical timing, enhance postoperative monitoring, and prevent complications based on PNI stratification. Furthermore, automatic PNI calculation and early warning functions should be embedded in electronic medical record systems to improve screening efficiency. Finally, challenges should be considered during implementation: differences in testing standards among different medical institutions may affect the universality of thresholds, and PNI needs to be used in conjunction with other prognostic indicators to avoid over-reliance on a single indicator. Future research should focus on prospective studies to validate the effectiveness of PNI-guided intervention pathways in improving clinical outcomes and to explore their suitability in regions with uneven healthcare resources.

## 6 Conclusion

As a clinically accessible, inexpensive, and noninvasive nutritional marker, PNI can effectively predict postoperative all-cause mortality and AKI in patients undergoing CABG. It is helpful for early identification of high-risk patients with poor prognosis, enabling targeted preventive and treatment measures. However, considering the inevitable heterogeneity and potential publication bias in this study, large-scale multicenter prospective cohort studies are needed in the future to evaluate the predictive value of PNI for prognosis after CABG, as well as to identify its influencing factors.

## Data Availability

The original contributions presented in the study are included in the article/Supplementary Material, further inquiries can be directed to the corresponding author.
