# Supplementary material for: Association between prognostic nutritional index and prognosis of patients receiving coronary artery bypass grafting surgery: a systematic review and meta-analysis
Source: Front Cardiovasc Med. 2026 Feb 19;13:1673038. doi: 10.3389/fcvm.2026.1673038 (PMC12960480; doi:10.3389/fcvm.2026.1673038)
Supplement: Supplementary file 1 [file Table1.docx]

TableS1 Literature search strategy

Pubmed-76

(("Coronary Artery Bypass"[Mesh]) OR ((((((Coronary Artery Bypasses) OR (Coronary Artery Bypass Grafting)) OR (Coronary Artery Bypass Surgery)) OR (Aortocoronary Bypass)) OR (Aortocoronary Bypasses)) OR (CABG))) AND ((prognostic nutritional index) OR (PNI))

Embase-45

((Coronary Artery Bypass or (Coronary Artery Bypasses or Coronary Artery Bypass Grafting or Coronary Artery Bypass Surgery or Aortocoronary Bypass or Aortocoronary Bypasses or CABG)) and (prognostic nutritional index or PNI)).af.

Cochrane-0

((Coronary Artery Bypass or (Coronary Artery Bypasses or Coronary Artery Bypass Grafting or Coronary Artery Bypass Surgery or Aortocoronary Bypass or Aortocoronary Bypasses or CABG)) and (prognostic nutritional index or PNI)).af.

Web of Science-23

((Coronary Artery Bypass) OR ((((((Coronary Artery Bypasses) OR (Coronary Artery Bypass Grafting)) OR (Coronary Artery Bypass Surgery)) OR (Aortocoronary Bypass)) OR (Aortocoronary Bypasses)) OR (CABG))) AND ((prognostic nutritional index) OR (PNI)) (Topic)
